# Selfish partners: resource partitioning in male coalitions of Asiatic lions

**DOI:** 10.1093/beheco/arx118

**Published:** 2017-09-25

**Authors:** Stotra Chakrabarti, Yadvendradev V Jhala

**Affiliations:** 1 Department of Animal Ecology & Conservation Biology, Wildlife Institute of India, Chandrabani, Dehra Dun, Uttarakhand 248 001, India

**Keywords:** behavioral plasticity, carnivore behavior, coalition, dominance hierarchy, mating skew, sociality

## Abstract

Behavioral plasticity within species is adaptive which directs survival traits to take multiple pathways under varying conditions. Male–male cooperation is an evolutionary strategy often exhibiting an array of alternatives between and within species. African male lions coalesce to safeguard territories and mate acquisition. Unique to these coalitions is lack of strict hierarchies between partners, who have similar resource securities possibly because of many mating opportunities within large female groups. Skewed mating and feeding rights have only been documented in large coalitions where males were related. However, smaller modal prey coupled with less simultaneous mating opportunities for male Asiatic lions in Gir forests, India would likely result in a different coalition structure. Observations on mating events (*n* = 127) and feeding incidents (*n* = 44) were made on 11 male coalitions and 9 female prides in Gir, to assess resource distribution within and among different sized male coalitions. Information from 39 males was used to estimate annual tenure-holding probabilities. Single males had smaller tenures and appropriated fewer matings than coalition males. Pronounced dominance hierarchies were observed within coalitions, with one partner getting more than 70% of all matings and 47% more food. Competition between coalition partners at kills increased with decline in prey size, increase in coalition size and the appetite states of the males. However, immediate subordinates in coalitions had higher reproductive fitness than single males. Declining benefits to partners with increasing coalition size, with individuals below the immediate subordinates having fitness comparable to single males, suggest to an optimal coalition size of 2 lions. Lions under different competitive selection in Gir show behavioral plasticity to form hierarchical coalitions, wherein partners utilize resources asymmetrically, yet coalesce for personal gains.

## Teaser

For a male lion, teamwork pays off but only to an extent. Using behavioral observations, we show that male Asiatic lions team-up to harness resources effectively, but form hierarchies unlike their egalitarian African cousins. Team members prosper better than loners in gaining and retaining access to females. However, teams of 2 males are optimum as low-ranking members of larger teams fare as poorly as loners. A hitherto unknown behavioral variation in lions highlights the flexibility of group living within species.

## BACKGROUND

Cooperation among males is an evolutionary strategy to enhance fitness of partners through a better defense of resources and reproductive opportunities (Krebs and Davies 1987). Such a strategy has been reported in diverse mammalian species like lions *Panthera leo* (Schaller 1972; Bertram 1975b; Packer and Pusey 1987; Meena 2009), cheetahs *Acinonyx jubatus* (Caro and Collins 1987), striped hyenas *Hyaena hyaena* (Wagner et al. 2008), chimpanzees *Pan troglodytes* (Nishida 1994; Watts 1998; Mitani et al. 2000), howler monkeys *Alouatta seniculus* (Pope 1990), baboons *Papio spp.* (Smuts 1985; Bercovitch 1988; Noë 1994), feral horses *Equus caballus* (Feh 1999), meerkats *Suricata suricatta* (Doolan and Macdonald 1996), coastal river otters *Lutra canadensis* (Blundell et al. 2004), and bottlenose dolphins *Tursiops truncatus* (Connor et al. 1992). Yet, the degrees of cooperation among male partners vary dramatically between species, from simple alliances in feral horses (Feh 1999) and coastal river otters (Blundell et al. 2004) to complex coalitions in nonhuman primates (Harcourt 1992). Loose alliances may be formed to gain “mutualistic benefits from simple aggregations” (Olson and Blumstein 2009) such as: extravigilance and predator protection in Cape ground squirrels *Xerus inaurus* (Waterman 1997), enhancement of hunting success in coastal river otters (Blundell et al. 2004) and effective defense of clumped resources in golden jackals *Canis aureus* (Macdonald 1979). But complex coalitions in which male partners incur costs-of-sharing valuable resources (like food, mates, and territory) seem to challenge Darwin’s (1859) theory of natural selection (Clutton-Brock 2009), wherein all individuals are supposed to compete for survival and reproduction, and not aid each other at their own costs. A typical coalition is defined as cooperation between 2 or more individuals against a third party during a competitive encounter (Harcourt 1992; Olson and Blumstein 2009; Koykka and Wild 2016). Such cooperation is potentially costly for the donors and tends to decrease their apparent fitness (Smith et al. 2010). Coalition formation in males can be explained through three major evolutionary pathways: 1) kin selection, where cooperation is extended to closely related individuals to enhance inclusive fitness of donors and recipients through shared genes (Smith 1964; Hamilton 1964); 2) reciprocal altruism, where cooperation improves the chances of future benefits between partners (Trivers 1971; Packer 1977); and 3) selfish support, which provides immediate benefits to the donor (Wrangham 1982) (for e.g., male chimpanzees act selfishly while helping nonkins against certain opponents to enhance their own dominance status, de Waal and Harcourt 1992). Such complex pathways for formation of coalitions necessitate species to be long lived, with frequent interactions between individuals and an ability to recollect past interactions (Ridley et al. 2005). Coalitions are thus, essentially found in highly social and cognitively developed species (Olson and Blumstein 2009), although cognitive restrictions on coalition formation have been debated recently (Bissonnette et al. 2014). Coalitions also show considerable variation within species, with recent literature suggesting competition and resource heterogeneity to be the major drivers of such differences (de Silva et al. 2016; Connor et al. 2017).

Other than nonhuman primates, the most well studied male coalitions are in African lions where groups of males aggressively compete to gain and preserve control over female prides (Schaller 1972; Bertram 1975b; Bygott et al. 1979; Packer and Pusey 1982; Grinnell et al. 1995). Only a few coalitions are able to take over territories and safeguard them for durations sufficiently long to sire one to several cohorts of cubs to full independence (Schaller 1972; Bertram 1975b; Pusey and Packer 1994). A high percentage of cubs fall victim to infanticide by new males during pride takeovers (Schaller 1972, Bertram 1975b; Packer and Pusey 1983a, 1983b; Packer et al. 1988; Banerjee and Jhala 2012). Akin to developed primates in lifespan, cognitive abilities and social bonding, the uniqueness about lions is the absence of dominance hierarchies between like sexes in their societies (Schaller 1972; Bertram 1975b; Bygott et al. 1979; Packer and Pusey 1982; Packer and Pusey 1985; Packer et al. 1988). Literature suggests that all adult pride females have equal opportunities to reproduce unlike in other carnivore societies like canids and hyaenids (Schaller 1972; Bertram 1975b; Packer and Pusey 1983b), and resource utilization is symmetrical between male coalition partners, with each male appropriating approximately equal feeding and mating opportunities (Bertram 1975b; Bertram 1978; Bygott et al. 1979; Packer and Pusey 1982, 1983b). The possible mechanisms giving rise to such a state of equal rights among male partners have been attributed to 2 factors: 1) frequent presence of large bodied prey in the African system, reducing the costs of sharing a meal (Funston et al. 1998), and 2) large number of simultaneous mating opportunities because prides in the African Serengeti comprise of an average of 6 (range: 2–18) adult females which are reported to exhibit synchronous estruses (Schaller 1972; Bygott et al. 1979; Packer and Pusey 1983a; Packer et al. 1988). The latter has been reported to release competition between males over ownership of receptive females (Bertram 1975b; Bygott et al. 1979; Koykka and Wild 2016). Additionally, reproduction in lions is highly inefficient, with an average requirement of about 1000 copulations which span across many mating events for a litter to be born (Bertram 1978). Thus, it is beneficial for a male lion to consort a single female for the entire estrous duration (2–6 days, Schaller 1972) to maximize chances of successful fertilization, leaving his other partners a chance to mate with other females, also most likely in estrus synchronously (Bertram 1975b). This has led to a scenario where coalition partners share their mating rights with remarkable equity, with no male being involved in more than 22% or less than 9% of all mating events (Bygott et al. 1979). However, competition for food and mates is more intense within very large coalitions and reduced only by kin selection, as males in such coalitions are usually closely related (Packer et al. 1991). In such coalitions mating is skewed with few partners being restrained from reproduction and thus, effectively acting as nonbreeding helpers (Packer et al. 1991). However, these males increase the overall fitness of the coalition through group protection (Packer et al. 1991).

Lions inhabit varied ecosystems which differ widely in resource availability (Van Orsdol et al. 1985). Asiatic lions (*Panthera leo persica*), now found only in the Gir forests of Gujarat, Western India, exhibit a social system wherein: prides essentially comprise only of females and their dependent cubs, while adult males live their lives separately, alone or in coalitions (Joslin 1973; Chellam 1993; Jhala et al. 2009; Meena 2009). Males encompass one-to-many female prides but are not an integral part of any particular pride. Interactions between males and female groups are limited mostly to matings with receptive lionesses and infrequent congregations on large kills (Meena 2009; Banerjee 2012). Male lions being subject to resource and sexual selection are expected to show behavioral plasticity in response to variations in the availability of prey and females (Krebs and Davies 1987). Male Asiatic lions likely undergo selective mechanisms different from their African Serengeti counterparts since their modal prey size (chital *Axis axis*, averaging at around 45 kg) is much smaller (Meena et al. 2011; Chakrabarti et al. 2016) compared to African systems (Hayward and Kerley 2005). Also, female prides of Asiatic lions are smaller, averaging at 2 adult females (Meena 2009; Banerjee 2012) which often lack estrous synchrony (present study), leading to less simultaneous mating opportunities for males. Since functional hierarchies within groups are shaped by competition (de Silva et al. 2016), we hypothesize that these limited resources should set the stage for enhanced competition between coalition males. Thus, if male partners in a coalition had differential abilities then it would result in a definitive hierarchy in terms of resource appropriation between them. We examine this possibility through continuous monitoring and observations on predation and mating events of free-ranging Asiatic lion coalitions of varying size (coalitions of 1–4 males). Our results indicate strong dominance-hierarchies between coalition partners, with pronounced asymmetry in resource utilization between them, indicating functional responses of behavior to changing resource availability. Such a hierarchical system was found both in small and large coalitions. Given such unequal sharing within coalitions, with subordinate males having inferior resource securities, we investigate the probable ultimate-causes of coalition formation in Asiatic lions. We postulate that although subordinate males get lesser resources, yet they would benefit directly from coalescing and should have higher reproductive success compared to single males.

## MATERIALS AND METHODS

### Ethics statement

All permissions to carry out field work were obtained from the Office of the Chief Wildlife Warden (CWLW), Gujarat under the provisions of the Wildlife Protection Act, 1972 (permit number: WLP/28/C/97–99/2011–16). Radio-collaring of lions was approved by the Ministry of Environment, Forests and Climate Change (MoEFCC), India (permit number: 22–7/2002 WL-I) and CWLW, Gujarat (permit number: WLP/26/B/356–61), and carried out under the supervision of field veterinary officers. Gir lions are quite accustomed to people on foot and in close proximity (Divyabhanusinh 2005; Banerjee et al. 2013) and behavioral observations on the individuals were done only after prolonged acclimatizing to our presence. Such habituations allowed us to observe them from as close as 20 m without hindering their daily behavioral repertoires.

### Study site and population

Between December 2012 and December 2016, 70 adult lions (21 males and 49 females) belonging to 11 coalitions and 9 prides were studied, encompassing an area of about 1200 km^2^ in the western part of the Gir Protected Area (Gir PA hereafter) and its adjoining human-dominated landscape (21°17′-20°55′N and 70°20′ - 70°52′E) in Gujarat, India. The study animals were a subset of the larger lion population in Gir PA (1800 km^2^) of around 250 individuals, which have been studied continuously since 1995 (Jhala et al. 1999, 2004, 2006; Meena 2008; Jhala et al. 2009, Banerjee and Jhala 2012; Banerjee 2012; Jhala et al. 2016). The intensive study area comprised of parts of the western Wildlife Sanctuary and the central National Park, and parts of the south-western agricultural landscape which is outside the formal boundaries of the PA. Gir PA is a dry-deciduous forest tract characterized by a semiarid climate (Champion and Seth 1968) with *Tectona grandis*, *Anogeissus spp.*, *Acacia spp.* and *Ziziphus spp.* as the dominant vegetation (Singh and Kamboj 1996; Jhala et al. 2009, Banerjee et al. 2013). The stretch outside the PA comprised mainly of farmlands, croplands, mango-orchards and *Prosopis spp.-Acacia spp*. thickets.

### Selection of coalitions

Males were categorized to be in a coalition when they were frequently seen in each other’s company, shared kills, hunted, vocalized and patrolled their territories together (Schaller 1972). Due to long-term research and intensive monitoring system in the study area since early 1990s (Chellam 1993; Jhala et al. 1999, 2004, 2006; Meena 2008; Jhala et al. 2009, Mena 2009; Banerjee and Jhala 2012; Banerjee 2012; Banerjee et al. 2013; Jhala et al. 2016), many lions were individually identifiable along with information on their ranging patterns and life histories. Using this prior information, territorial male coalitions: 1) of varying sizes, and 2) with information since they became residents in the area were selected. We chose coalitions with neighbouring ranges as coalitions dispersed over a very large area were difficult to monitor simultaneously with intense rigor. A total of 11 coalitions comprising of singletons/single male (*n* = 4), doubletons/2-male coalitions (*n* = 5), more than 3 male coalitions (*n* = 2) and their interacting 9 female prides (*n* = 49 adult females) were selected for behavioral observations and were monitored for periods ranging between 1.5 and 4 years.

### Identification and monitoring

Study individuals were uniquely identified using their vibrissae patterns and additional body marks (Pennycuick and Rudnai 1970; Jhala et al. 1999). A combination of radio-telemetry and intensive search using cues such as pugmarks, prey-alarm calls, roars, kills, and information from tourists were used to track and monitor the individuals. Two adult individuals (1 male belonging to a coalition of 4 males and 1 female belonging to a pride of 3 adult females) were radio-collared (GPS collars, Vectronics Aerospace GmbH, Berlin, Germany, weighing less than 1% of the animal’s bodyweight). The entire monitoring period of each male was divided into 2-day sampling occasions as mating observations necessitated each male to be visually located at least once in 2 days, so as not to miss recording a mating event (lion mating events typically range from 2 to 6 days, Schaller 1972; Bertram 1978; Packer and Pusey 1983a). Such intensive monitoring was possible owing to rigorous fieldwork aided with an age-old practice of the forest department to track individual lions every day (Singh and Kamboj 1996; Divyabhanusinh 2005; Meena and Kumar 2012). Our efforts led to the detection of each male in 92 ± 1% of all the sampling occasions (Supplementary Table S1). All the study individuals were familiar to our presence, and were followed on foot or a 4-wheel drive vehicle.

### Behavioral observations

#### Mating events

Mating events were recorded by locating each study male every day or every alternate day. Upon locating a male, the GPS coordinates, surrounding habitat, state of activity and associated animals were noted. One mating event was considered to be the entire duration when a male consorted a lioness in estrus (included the initial courting phase, actual copulations and intervals between successive copulations, see Supplementary Figure S1 for details) till the pair parted ways and returned to their respective groups. Once a mating pair was found, the male and female were identified to their coalition and pride respectively, and a continuous 24-h focal behavior sampling (Altmann 1974) was done for all days the mating event lasted. Pairs were kept in view within 50 m from observers day and night. During dark nights a flash light was used every 15–30 min to ascertain location of the mating pairs and copulations outside visible range were confirmed with the distinctive loud “*yowl*” that males make while ejaculating (Schaller 1972; Bertram 1978). Total mating durations and partner-switching instances were recorded. For computing mating durations, we used only those events (*n* = 119/127) where we could observe pairs from the beginning of the events (courting phase). Since study coalitions differed in their total monitored durations (depending upon their initiation of residence/being territorial in the area), to remove bias emanating from differential sampling efforts, number of mating events of a male was expressed as a ratio to the number of days the male was actually detected in the field. Also, we attempted to locate study males once in each of the sampling occasions (2 days), but we failed to detect them in a few cases (8%). Thus, there were chances that we could have missed mating events and the above mentioned calibration addresses this problem. For each male, calibrated mating frequency was expressed per year and this mating index (MI = [number of mating events/number of days detected in field] × 365] was then compared between partners and tested for differences using a chi-square test at an α value of 0.05.

#### Feeding events

**Figure 1:**
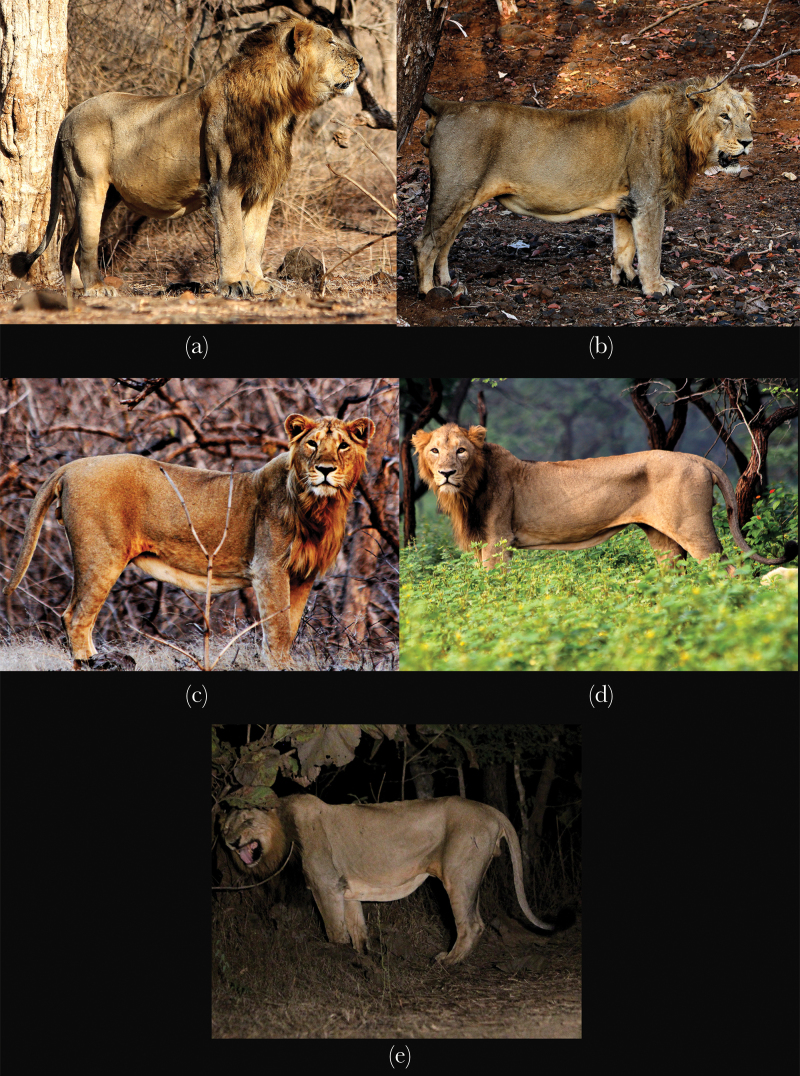
Belly scores to determine the state of hunger/appetite of individual male lions following Bertram (1975a): (a) Fully gorged with a bloated belly, belly fold taut and almost invisible, scored as 1; (b) Well-fed individual with a distended belly and a hint of the belly fold seen underneath, scored as 2; (c) Belly line almost parallel to the ground with a prominent belly fold, animal not too fed, neither too starved, scored as 3; (d) Semistarved individual with a very prominent fold and hints of lateral pelvic depressions, scored as 4; (e) Fully starved individual, with a very loose belly fold and prominent lateral depressions, scored as 5. Photographs were taken by first author.

Feeding behavior of coalition partners was recorded from the beginning of a feeding event (when the males started feeding on a kill) to the full utilization of the carcass (when the males permanently left it). Data were used from only those events (*n* = 44) where initiation of feeding was known with certainty and ≥2 males were present at the site, within 100 m of the carcass. We postulated that competition at kills and hence dominance-hierarchies, if any, would depend upon: 1) prey size, 2) appetite state/hunger of the males, and 3) number of individuals sharing a kill. Prey weights were visually estimated. Before collecting data in the field, we practiced and compared our prey weight estimating skills by accurately weighing different sized whole carcasses used for feeding trials on lions in a zoo facility (Chakrabarti et al. 2016). We could accurately estimate weights of small carcasses up to 15 kg (with an error of ± 1 kg) and medium carcasses up to 100 kg (with an error of ± 5 kg). Visual estimates of very large carcasses (>200 kg) differed slightly among observers and hence a consensus weight between 2 to 3 observers was taken for such prey in the field. The appetite state of every male lion was recorded for each event by scoring their belly sizes following Bertram’s (1975a) technique for African lions. Each lion was given a belly score between 1 (fully gorged) and 5 (starved) (detailed in Figure 1). Information regarding the feeding sequence (males taking turns or feeding simultaneously) and aggression at kills was documented. Total time spent by each male feeding on a carcass was recorded through continuous 24-h monitoring of the feeding events for all days a carcass was being fed upon. Akin to mating observations, each carcass was kept in sight and night monitoring was done using flashlights. Feeding durations were taken as surrogates of biomass consumption. However, lions (like other carnivores) tend to selectively feed first on the choicest body parts of prey (visceral organs and flesh, which need very low handling time), and then the less digestible body parts like skin, bones, and hide, which require considerably higher handling durations (Chakrabarti et al. 2016). Consequently, a male eating first would consume more of higher quality food in relatively less time feeding on viscera and flesh than the next ones having to negotiate skin, bones, and hide. Thus, using absolute feeding duration alone would not account for quality and amount of consumption. To circumvent this problem, we used data (from feeding trials on wild-caught lions which mimicked free-ranging conditions, Chakrabarti et al. 2016) on consumption rates (kg eaten/h) of lions for successive days feeding on the same carcass. Whenever male partners fed sequentially from small–medium carcasses (<100 kg) in the wild, a correction factor of 0.53 (=consumption-rate ratio of 2^nd^ to the 1^st^ day in the captive trials, Chakrabarti et al. 2016) was multiplied to the feeding time recorded for males eating second, third and so on. For larger carcasses (>100 kg), the correction factor was used for males eating after 12 h from the initiation of feeding. The disparity in consumption between partners was then calculated as the difference in *corrected feeding time* on a kill. Also, aggressive behavior between the partners on a kill (a measure of competition) was categorized into 2 classes: 1) aggressive exclusion—when the feeding male(s) thwarted the advance of at least one of his (their) partners through heightened aggression and did not allow him (them) to feed, and 2) meal sharing—mild aggression between partners (squabbles and occasional swats), but all partners shared a kill simultaneously.

We examined whether difference in consumption between partners was significantly different from zero using a one-tailed *t* test, expecting a significant positive difference in consumption between male partners. The difference (if significant) was then modelled with estimated prey size, number of males at the site/coalition size and the appetite state of the males. We expected pronounced competition (hence dominance) at smaller kills with greater number of “hungry” partners at the kill site. We tested 4 models bearing additive as well as interactive effects of prey size, appetite state of males (belly scores) and coalition size against the null model. We ranked models using Akaike Information Criterion corrected for sample size (AICc) (Akaike 1974) and significance levels, and assessed their goodness-of-fit using R^2^ statistic and residual diagnostics.

### Fitness quotient

Staying alone or forming coalitions are alternative survival/reproductive strategies for males in social mammals, including lions (Smuts 1985; Pope 1990; Bygott et al. 1979; Feh 1999). However, in African lions, males in coalitions are more successful than singletons, producing more number of offspring (Bygott et al. 1979). For coalitions to evolve as a strategy: 1) coalitions should be able to secure more resources compared to singletons, and b) if dominance-hierarchies are present within coalitions, then subordinate members should also get higher benefits than males which do not form coalitions, especially if coalition partners are unrelated. To test this postulate, we compared reproductive fitness of singletons with those that form coalitions. Since it was difficult to enumerate the number of actual surviving offspring of individual males in the wild with certainty, we used 2 parameters to index reproductive fitness of males: 1) tenure holding ability: tenure length is an important facet of lifetime-success as reproductive fitness of male lions depends upon their ability to acquire and defend territories (Packer et al. 1988), and 2) mating index of each male: as a surrogate for the number of offspring produced, assuming higher chances of successful fertilization with more matings.

*Fitness quotient of a male = Annual tenure holding probability × Mating index*

Annual and span tenure-holding probabilities of adult males belonging to different coalition sizes (1, 2, and >2) were computed using a known-fate model as the fate of the males were known with certitude (similar to computing survival probability using Kaplan-Meier estimator, Williams et al. 2002; Skalski et al. 2005) in program MARK (White and Burnham 1999). Since the date of tenure-acquirement was known with certainty to the month for each of the coalitions used in this analysis, and owing to limited sample sizes and similar conditions spanning our study period, we did not test for the effect of different time periods on coalition tenures. Instead, for computing tenure-holding probabilities we considered all of the study coalitions to have commenced their tenureships contemporaneously. The weekly observations on coalition survival were pooled for a month which was used as the minimum unit for this analysis. Some coalitions continued to hold tenures at the end of this study and they were right censored. Subsequent analysis provided monthly survival probabilities from which annual probabilities were derived for different sized coalitions. For this analysis, in addition to the 21 males (in 11 coalitions) monitored for behavioral observations (see the section “Selection of coalitions” for details), we also used information from males (*n* = 18 in 10 coalitions) monitored between 2004 and 2011 (Jhala et al. 2006 and Jhala et al. 2011). Data from a total of 8 singletons, 9 doubletons, and 4 coalitions with >2 males were analyzed. Fitness quotients were then compared between coalitions.

All data processing was done using MS Excel and analyses using program R v15 (R Core Team 2013) and MARK (White and Burnham 1999).

## RESULTS

### Behavioral observations

#### Mating events

**Figure 2:**
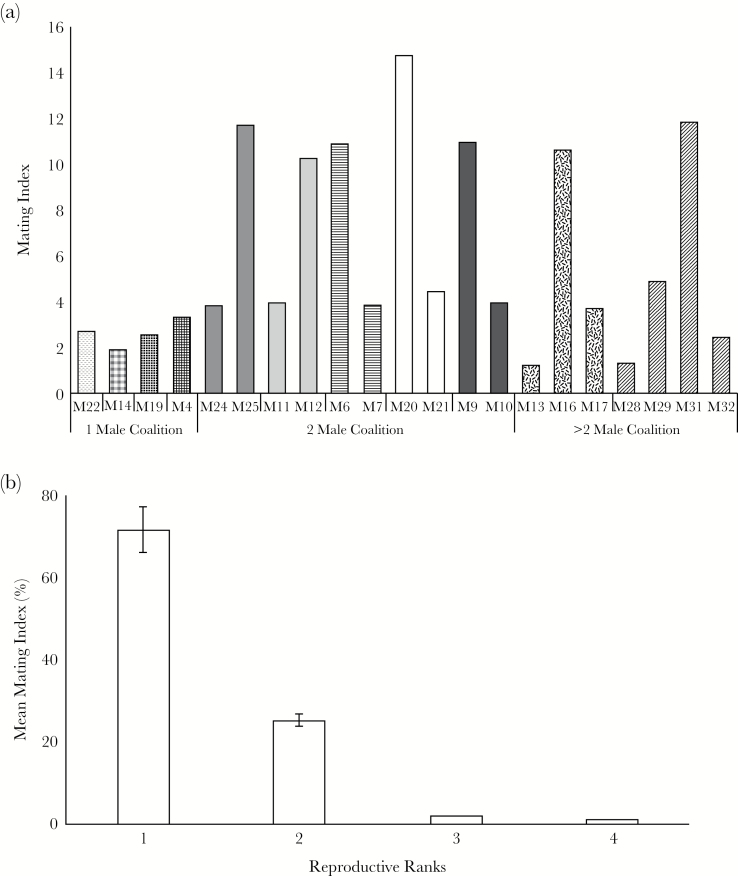
Distribution of observed mating events within and between coalition males. Plots showing: (a) Mating index of monitored lions (annual mating frequency calibrated by the total number of days each male was detected in the field), adjacent bars with similar patterns represent lions from the same coalition; and (b) Lions were ranked in a descending order of mating index within each coalition. The figure shows percent matings procured by lions within a coalition averaged for each rank across coalitions. Error bars represent 95% CIs.

We recorded 127 mating events and invested 9305 h of focal sampling for collecting observational data. Male–female mating association lasted for an average of 72.9 ± 2.8 h. Also, in only 1% (2 out of 127 events) of all the recorded mating events we found another female of the same pride in estrus synchronously. When compared between partners within a coalition, mating indices differed significantly (χ^2^ = 41.22, df = 16, *P* = 0.0005), with one male being consistently involved in more matings than his partner(s) (Figure 2a). Skew in the distribution of mating events between partners was highly conserved among different coalitions. The partners with most matings appropriated 71.6 ± 3%, the partners with next-highest matings had 25.3 ± 1% and the partners with least matings had 1–2% of the total events of their respective coalitions (Figure 2b).

#### Feeding events

Data from feeding events of free-ranging lion coalitions revealed a similar trend as found from mating observations. Biomass consumption was highly skewed (difference in consumption between partners > 0, one-tailed *t* = 6.06, df = 43, *P* < 0.001) and the reproductively dominant males consumed 0.47 ± 0.07 times more from kills than their partner(s). This difference in consumption was best explained by a 3 parameter linear model (GLM of the Gaussian family) having the additive effects of prey size, appetite state of the male with highest matings (reproductively dominant) in the coalition and the number of males at the kill site/coalition size (*R*^2^ = 0.48, df = 5, *P* < 0.001, Supplementary Table S2, Supplementary Figure S2). The model was given by:

*Difference in biomass consumption = −1.045(±0.331) − 0.002(±0.0005) × prey size + 0.313(±0.091) × coalition size + 0.312(±0.083) × belly score* [figures in parentheses represent SEs]

We recorded high levels of aggression between partners which increased with decline in prey size, increase in number of partners at the kill site and their appetite states (Supplementary Figure S3). Dominant males aggressively excluded other partners and consumed 47% more from shared kills. This further indicated that above-mentioned variables were important in parameterizing feeding hierarchies. However, none of the interaction terms were significant and hence were not included in the best model which differed from the next best model by a ∆AICc > 9 (Supplementary Table S2).

### Fitness quotient

**Figure 3:**
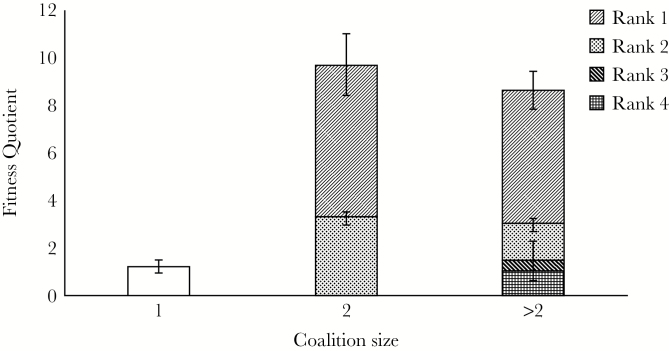
Reproductive-fitness quotients of male lions in different sized coalitions. Error Bars represent 95% CIs.

Singletons held territories for shorter durations (annual tenure holding probability = 0.47 ± 0.19) than males in coalitions. Coalitions of 2 males and more than 2 males had similar annual tenure-holding probabilities (0.85 ± 0.05 and 0.81 ± 0.07, respectively). Singletons had far lower fitness quotients than subordinate males in a coalition of 2 (Figure 3). However, in coalitions with more than 2 males, the males at the bottommost ranks (rank 3 and below) had fitness comparable to that of singletons, indicating that they would do equally good (*or poorly*) if they remained alone.

## DISCUSSION

Functional responses of behavior to different drivers of selection are crucial for individual fitness. Plasticity in strategies aid individuals in coping with varying environmental conditions (Krebs and Davies 1987). Male cooperation to form coalitions is one such strategy which exhibits a wide array of inter- and intraspecific variation in mammals. Coalition formation can vary within species depending upon habitat and resource heterogeneity (Connor et al. 2017). Using lions as model species, we demonstrate behavioral plasticity to be a possible function of resource availability. Male African lions in the Serengeti system have been found to cooperate amongst themselves to gain access to food and mates, but are not reported to form strict dominance hierarchies (Schaller 1972; Bertram 1975; Bertram 1978; Bygott et al. 1979; Packer et al.1988). Asiatic lions, living in more forested habitats with smaller modal prey and less simultaneous mating opportunities, likely face selective pressures that results in pronounced dominance hierarchies within male coalitions.

Our results indicate that in male Asiatic lions mate and food sharing between coalition partners were highly skewed. One of the males in every coalition was consistently involved in more matings and the same individual got the *lion’s share* from kills compared to his partner(s). As postulated, competition at kills was high amongst partners, very prominent at small carcasses, with high appetite state of the dominant males and more partners in a coalition. A distinct feeding order was observed among the partners, where they took turns to eat from relatively smaller carcasses. The reproductively dominant males invariably had the first rights to carcasses, even if they were not the killers or first possessors. However, dominant partners were observed to share small kills amicably with their partners when the former had their bellies full (Supplementary Figure S3). We also recorded 3 instances of intra-coalition mate switching where the female switched from one male to its coalition partner within the same estrous duration. In all of the 3 cases the switch happened in favor of the male who also appropriated the maximum mating opportunities and food at kills within that coalition. Reproductive dominance across different ranked individuals within coalitions was found to be highly preserved among coalitions, with males at the bottommost ranks hardly getting any matings (Figure 2b). Thus, in an Asiatic system, individuals in large coalitions (3–4 males) have very asymmetrical resource securities, which might be a plausible explanation of such coalitions being rare. Our results primarily indicate that although male coalitions exhibit pronounced hierarchies, immediate subordinates are better off (higher fitness) than single-males. We predict an optimum coalition size of 2 in male Asiatic lions, below and beyond which reproductive success of single males and low-ranking subordinates respectively are low. This is in accord with the ground reality of an average adult male group size of 2.1 ± 0.3 in Gir (Gogoi 2015). Our results further corroborate the findings of de Silva et al. (2016) where African and Asian elephant groups (*Loxodonta africana* and *Elephas maximu*s) show different hierarchical systems shaped by resource competition, and Connor et al. (2017) where male alliances of bottlenose dolphins exhibit considerable variation in habitats differing in resources and threats.

However, apparent reproductive fitness alone cannot explain coalition strength since in large coalitions (>2 males) lowermost ranked individuals had very low reproductive fitness, yet such coalitions exist. Other than mate and territory acquisitions, a coalition may also provide other direct benefits through group protection and food procurement. These may be vital for subordinate lions for survival, gaining vigor and subsequently attempt to either go up on the dominance ladder in the same coalition or join/form other coalitions, as reported in feral horses (Feh 1999). We have observed lions that have lost their coalition partners join other males to form new coalitions, sometimes differing widely in their ages. In African lions different aged coalition partners were mostly found in small coalitions and large coalitions were typically composed of similar aged closely related kins (Packer et al. 1991). Thus, genetic analysis of relatedness within different male Asiatic lion coalitions would shed more light on the underlying mechanisms of the observed patterns. Uniqueness of the observed social structure make Asiatic lions stand out as a distinct behavioral ecotype, highlighting plasticity of social behavior within species facing different selective pressures. Funston et al. (1998) record land tenure system of lions in Kruger to be similar to that found in the Asiatic system wherein males primarily safeguard territories which encompass one-to-many female prides. It would be interesting to see if a social structure similar to what we report for male Asiatic lions exists in Kruger and other lion systems of Africa where forested settings make males interact less with females with the latter living in smaller groups compared to that found in the East African plains.

## SUPPLEMENTARY MATERIAL

Supplementary data are available at *Behavioral Ecology* online.

## FUNDING

The work was supported by the Wildlife Institute of India and the Department of Science and Technology, India (Grant number: SERB/ F/0601/2013–2016).

## Supplementary Material

Supplementary_information

Figure_S1

Figure_S2

Figure_S3
