# Supplementary material for: Selfish partners: resource partitioning in male coalitions of Asiatic lions
Source: Behav Ecol. 2017 Sep 25;28(6):1532–9. doi: 10.1093/beheco/arx118 (PMC5873260; doi:10.1093/beheco/arx118)
Supplement: Supplementary_information [file arx118_suppl_supplementary_information.doc]

**Supplementary figures Legends**

**Figure S1.** Different behavioral-stages in a mating sequence of Asiatic lions: **a)** A male walks in tandem with a lioness in estrus, never leaving her unguarded or out of sight, **b)** Solicitation of copulation by the lioness, **c)** Mounting and copulation, with nape and ear biting movements by the male who yowls loudly at ejaculation, **d)** The lioness snarls semi-aggressively at this stage, and turns and swats at the male, **e)** The male follows the lioness persistently throughout her entire estrus period, often just steps behind, his nose almost touching her rear. Photographs taken by first author.

**Figure S2.** Residual-diagnostic plots of best model relating difference in biomass consumption between coalition partners to prey size, coalition size and the appetite state of the reproductively-dominant male.

**Figure S3.** Scatter plot showing how aggression between male coalition partners on a kill change with prey size, appetite state of the reproductively dominant partner (quantified through belly scores) and number of male partners at the kill (coalition size). Aggression between males increased with lower prey size, greater number of partners and higher appetite of the reproductively dominant males. **Empty circles**: *aggressive exclusion*, when feeding male(s) thwarted the advance of at least one of his (their) partners through heightened aggression and didn’t allow him (them) to feed; and **Filled circles:** *meal sharing*, mild aggression between partners (squabbles and occasional swats), but all partners fed on a kill simultaneously.

**Supplementary Tables legends**

**Table S1.** Sampling effort for monitoring male coalitions on which behavioral observations were made.

**Table S2.** Model selection statistics and parameter estimates of the best model relating difference in biomass consumed from kills by coalition partners to prey size, coalition size and appetite state of the reproductively dominant male (quantified through belly scores) in the coalition.

**Table S1**

| **Male id** | | **Coalition size** | **Monitored Occasions** | **Detected Occasions** | **Detection proportion (%)** |
| --- | --- | --- | --- | --- | --- |
| M22 | 1 | | 246 | 231 | 94 |
| M14 | 1 | | 196 | 174 | 89 |
| M19 | 1 | | 268 | 255 | 95 |
| M4 | 1 | | 214 | 184 | 86 |
| M20 | 2 | | 415 | 403 | 97 |
| M21 | 2 | | 415 | 394 | 95 |
| M6 | 2 | | 552 | 530 | 96 |
| M7 | 2 | | 552 | 513 | 93 |
| M11 | 2 | | 451 | 419 | 93 |
| M12 | 2 | | 451 | 428 | 95 |
| M9 | 2 | | 288 | 262 | 91 |
| M10 | 2 | | 288 | 251 | 87 |
| M24 | 2 | | 492 | 438 | 89 |
| M25 | 2 | | 492 | 423 | 86 |
| M28 | 4 | | 274 | 258 | 94 |
| M29 | 4 | | 274 | 249 | 91 |
| M31 | 4 | | 274 | 241 | 88 |
| M32 | 4 | | 274 | 219 | 80 |
| M13 | 3 | | 300 | 276 | 92 |
| M16 | 3 | | 300 | 287 | 96 |
| M17 | 3 | | 300 | 264 | 88 |

**Table S2**

| Model | n | ΔAICc | AICc | R^2^ | Int.(SE) | Pr.Size(SE) | Cln. Size(SE) | Appt.(SE) | Pr.Size*Appt. (SE) | Pr.Size*Cln.Size(SE) |
| --- | --- | --- | --- | --- | --- | --- | --- | --- | --- | --- |
| **Pr.Size+Cln.Size+Appt.** | **44** | **0** | **34.12** | **0.48** | **-1.045(0.331)** | **-0.002 (0.0005)** | **0.313(0.091)** | **0.312(0.083)** |  |  |
| Pr.Size+Appt. | 44 | 9.41 | 43.53 | 0.32 | -0.382(0.302) | -0.002(0.0005) |  | 0.323(0.093) |  |  |
| Pr.Size*Cln.Size | 44 | 11.33 | 45.15 | 0.32 | -0.588(0.412) | 0.002(0.002) | 0.534(0.176) |  |  | -0.001(0.001) |
| Pr.Size *Appt. | 44 | 11.39 | 45.51 | 0.31 | -0.421(0.421) | -0.001(0.003) |  | 0.334(0.126) | -0.0001(0.001) |  |
| Null | 44 | 21.85 | 55.97 |  | 0.462(0.066) |  |  |  |  |  |

Feeding events [n], Akaike Information Criterion corrected for sample size [AICc], Prey Size [Pr.Size], Coalition Size [Cln.Size], Appetite state of reproductively-dominant male [Appt.], parameters: mean
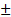
SE
