# Supplementary figures and images for: Selfish partners: resource partitioning in male coalitions of Asiatic lions
Source: Behav Ecol. 2017 Sep 25;28(6):1532–9. doi: 10.1093/beheco/arx118 (PMC5873260; doi:10.1093/beheco/arx118)

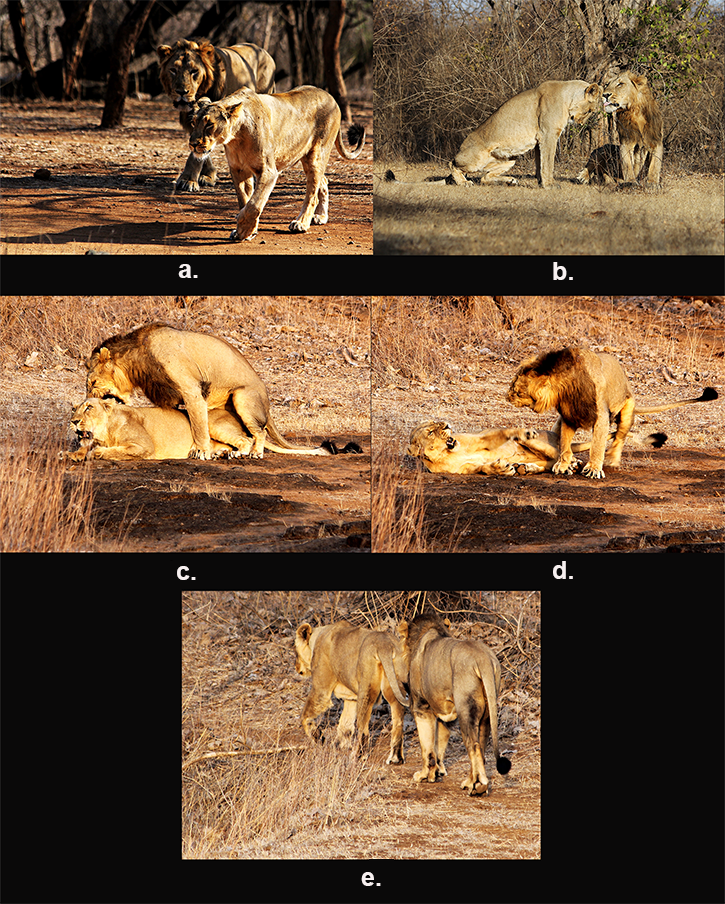

Supplement: Figure_S1 [file arx118_suppl_figure_s1.png]

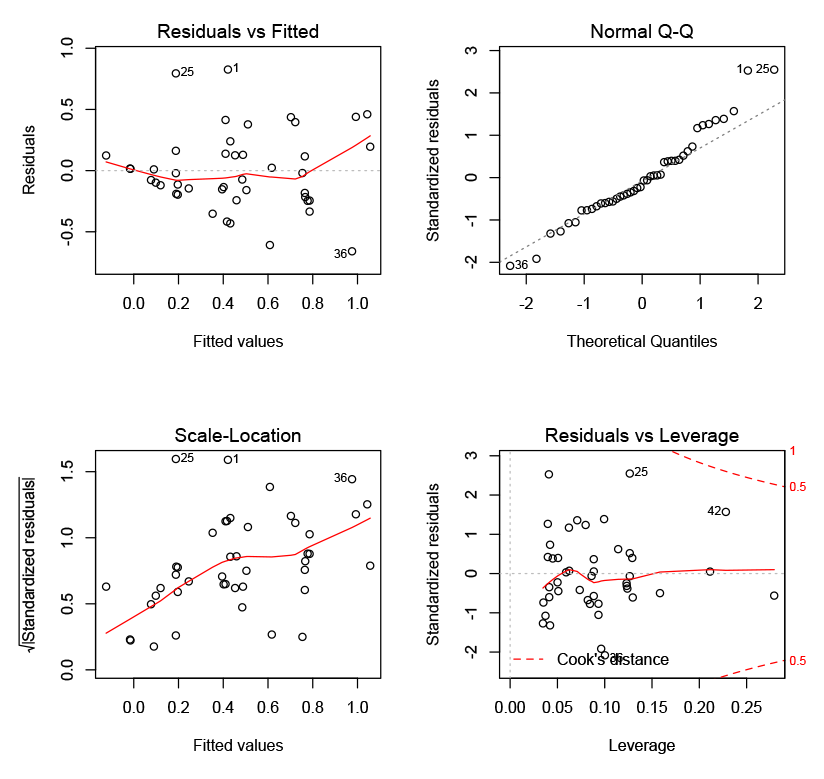

Supplement: Figure_S2 [file arx118_suppl_figure_s2.png]

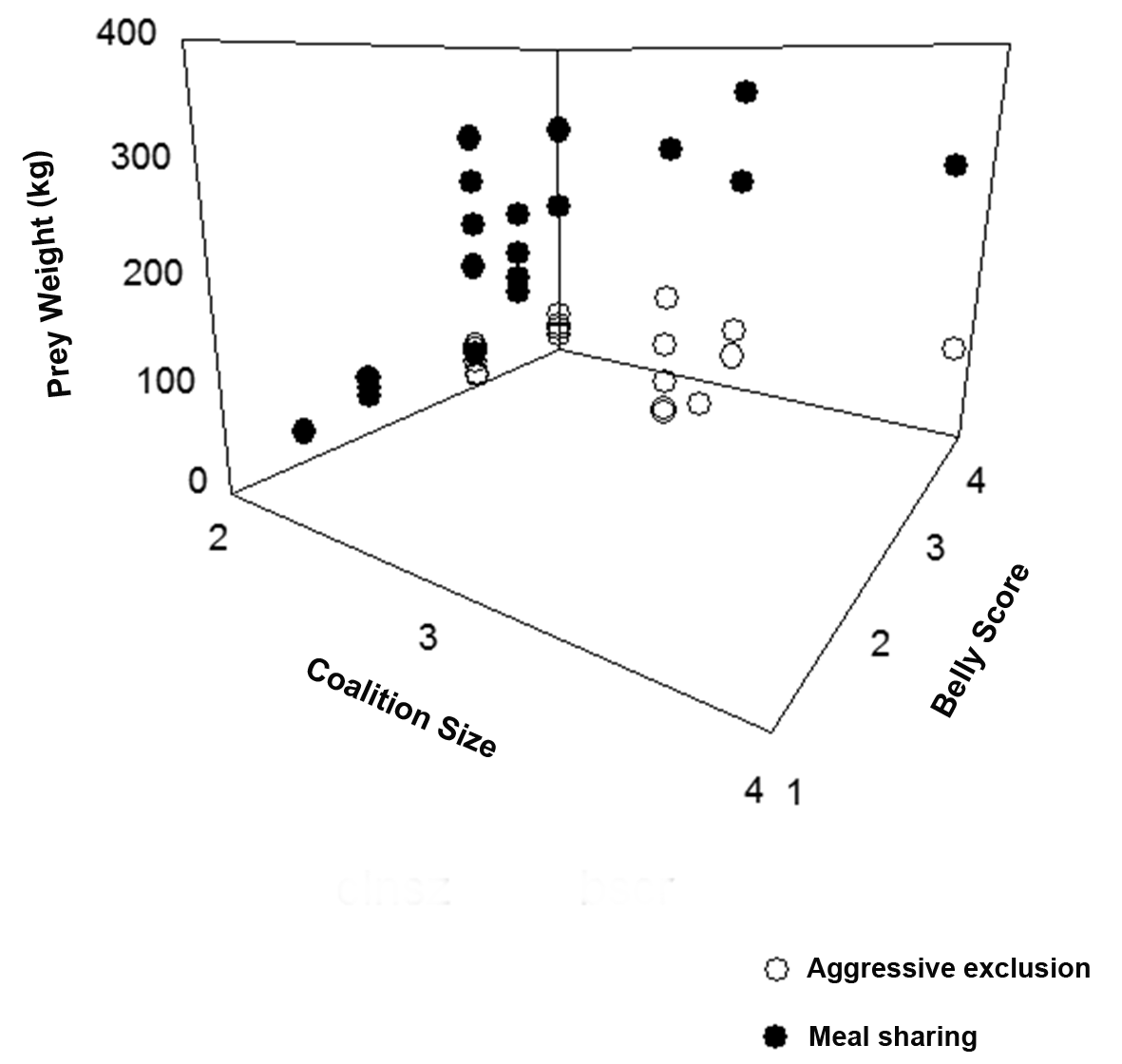

Supplement: Figure_S3 [file arx118_suppl_figure_s3.png]
